# ECT: Knowledge and attitude among patients and their relatives

**DOI:** 10.4103/0019-5545.31616

**Published:** 2006

**Authors:** B.S. Chavan, Suresh Kumar, Priti Arun, Chander Bala, Tushar Singh

**Affiliations:** * Professor and Head; ** Assistant Professor; *** Reader; **** Medical Social Worker; ***** Senior Resident

**Keywords:** Knowledge, attitude, ECT, questionnaire

## Abstract

**Background:**

It is believed that people lack sound knowledge and appropriate attitude towards electroconvulsive therapy (ECT). However, very little systematic research has gone into this area.

**Aim:**

To examine the knowledge and attitude of patients and their relatives towards ECT.

**Methods:**

A 16-item questionnaire with satisfactory face validity and content validity was constructed and translated into Hindi. It was then administered to 89 patients and 83 relatives attending the psychiatry services in a major hospital in north India.

**Results:**

More than 65% of the respondents in both the groups—patients as well as relatives—gave correct responses such as ECT is life saving, many times it causes temporary but not permanent memory impairment and that ECT is not a non-scientific treatment. There was non-significant disagreement between the two groups.

**Conclusion:**

The study is a preliminary exploratory one and is likely to give direction for further research with refined methodology.

## INTROUCTION

Despite evidence that electroconvulsive therapy (ECT) is effective^1^–^3^ and safe^3^, and leads to shorter and less costly inpatient treatment,^4^ it is rarely used as the first line of treatment and is generally reserved for older and resistant cases of depression and other psychiatric disorders. Certain factors such as social stigma, inadequate undergraduate training, doubts about its efficacy and safety, ambivalence among psychiatrists and doubts about its being a cost-effective alternative to antidepressant treatment, might have limited the use of ECT in the management of depression. It has been reported that the use of ECT is not uniform throughout the United States. While examining various reasons of limited use of ECT, Salzman has commented that ECT failed to attract scientists to carry out research on ECT as compared to antidepressant treatment. Much of the content in this paragraph is USA-sensitive.

Numerous studies have addressed the issue of knowledge and attitude towards ECT not only among the patients^6^–^10^ and their relatives^8^ but also among the lay public^12^, among adolescent patients and their parents,^13^,^14^ and also among health professionals including psychiatrists.^1^,^15^ Although no standard procedure for attitude assessment exists, most of these studies have utilized a questionnaire framed from the experience of clinicians. Concerns were frequently expressed, probably because ECT was not fully understood by the patients and their families.^13^ The authors of this study, too, noted from their clinical experience that patients and their relatives had many misconceptions as well as a negative attitude towards the use of ECT. Previous studies had taken up only those patients who had completed a course of ECT in past.^6^,^7^,^10^ A notable difference between the findings of western and Indian studies is that knowledge is quite satisfactory and the attitude is positive in Indian subjects.^10^,^15^–^18^ The present study was carried out to examine the knowledge and attitude towards ECT among patients and relatives attending the psychiatric services in a major hospital in north India.

## METHODS

The study was conducted at the Department of Psychiatry, Government Medical College, Chandigarh with the aim to study with the help of a questionnaire the knowledge and attitude of patients and their relatives towards ECT. The study was undertaken with the following objectives:

### Objectives

1. Construction of a questionnaire
2. Assessment of attitude and knowledge of stable psychiatric patients
3. Assessment of attitude and knowledge of healthy relatives of psychiatric patients
4. Comparison of responses between relatives and patients

The study was conducted in two phases

**Phase 1:** Framing of the questionnaire—The items for the questionnaire were drawn from two sources:

1. Published questionnaires and scales that assess attitudes of diverse populations towards ECT.
2. Clinical experience of three psychiatrists based on detailed interviews with both patients and their relatives who had been offered/advised ECT.

After a consensus among the three psychiatrists, an initial pool of 20 items was obtained. The questionnaire covered areas of efficacy, safety, frequency of use, indications for use, mode of action, side-effects and practical aspects of ECT administration. The subjects were given response choices of ‘agree’, ‘disagree’ and ‘uncertain’. The questionnaire was initially constructed in English, later translated into Hindi by three psychologists independently to obtain agreement on the translated version and the questionnaire was back translated into English. This English version was compared with the original English version to ensure content validity.

To get a rough estimate of the face validity, three senior faculty members (other than the three psychiatrists who constructed the questionnaire) independently scored each item as ‘right’ or ‘wrong’. Of the 20 items, there was 100% agreement on 16 items. Therefore, only those 16 items were selected for administration to the test population (Table 2).

**Phase 2:** Administration of the questionnaire to the study population: Patients and relatives were assessed individually by a social worker of the Department of Psychiatry on sociodemographic variables followed by evaluation on the 16-item questionnaire. The sample was drawn from the psychiatry OPD as well as ward from July 1996 to December 1996. Patients with acute disturbances (acute intoxication with alcohol or drugs, acutely psychotic), organic brain disorders and with mental retardation were excluded, i.e. only psychiatrically stable patients were recruited. Similarly, relatives with past or present psychiatric illness were excluded, i.e. only psychologically healthy relatives were included.

## RESULTS

### Sample characteristics

The sample characteristics of both patients and relatives are given in Table 1.

**Table 1:** Sample characteristics

Characteristics	Patients (*n*=89)	Relatives (*n*=83)
Age (years)	35.0 (SD 13.4, range 16-70)	37.0 (SD 14.8, range 16-70)
Males	52 (58.4%)	57 (68.7%)
Education (Matric and above)	41 (46.1%)	42 (50.6%)
*Diagnosis*		
Mood disorders	26 (29.2%)	
Psychotic disorders	7 (7.8%)	
Drug and alcohol-related disorders	17 (19.1%)	
Neurotic disorders	26 (29.2%)	
Others	13 (14.6%)	

### Tool (questionnaire) characteristics

The correct response for each item was predetermined. The response ‘agree’ was to be scored as correct for four items (1, 6, 7, 10) and the response ‘disagree’ was to be correct for all the remaining 12 items. There was no item with ‘uncertain’ as the correct response.

### Response tendencies

The patients and their relatives gave more or less similar responses (Table 2). Items depicting very good knowledge (defined arbitrarily as endorsed correctly by more than 70% respondents) were four in number (3, 7, 11, 15) while good knowledge (defined arbitrarily as correctly responded by more than 50% population) was seen on seven items (2, 6, 8, 9, 10, 13, 16). The patients as well as the relatives were found poor in knowledge and negativistic in attitude (defined arbitrarily as correct responses given by less than 20% of respondents) on one item (No. 1).

**Table 2:** Responses of patients and relatives on all items of the questionnaire

Item	Correct responses given by	
	
	Patients *n=89* (%)	Relatives *n=83* (%)
1. Pregnant women can also receive ECT	15 (16.8)	13 (15.7)
2. ECT is an inhuman treatment	54 (70.7)	45 (54.2)
3. Use of ECT leads to permanent loss of memory	79 (88.8)	77 (92.8)
4. ECT is given to only those patients who have little chance of improvement anyway	26 (29.2)	25 (30.1)
5. Following discovery of new medicines, treatment with ECT should not be done	38 (42.7)	39 (47.0)
6. Use of ECT leads to temporary impairment of memory	49 (55.1)	53 (63.9)
7. Many times ECT proves to be life-saving	76 (85.4)	75 (90.4)
8. Patients of epilepsy should not be given ECT	50 (44.9)	38 (45.8)
9. ECT is given as a punishment to violent/angry patients	53 (59.6)	53 (63.9)
10. ECT can be given to older persons also	48 (53.9)	49 (59.0)
11. There is no scientific proof favouring utility of ECT	71 (79.8)	58 (69.9)
12. ECT is painful	37 (41.6)	31 (37.4)
13. If ECT fails in a patient, then no other treatment will succeed	56 (62.9)	55 (66.3)
14. ECT should not be given more than once a week	25 (28.1)	31 (37.4)
15. There is no need of investigations before ECT	77 (86.5)	76 (91.6)
16. ECT should be given only to patients admitted in the ward	48 (53.9)	43 (51.8)

Tendency to choose different categories of responses was comparable in the two groups (patients and relatives). No particular response bias, such as tendency to agree and tendency to choose the first response, etc. was seen.

The response ‘Uncertain’ was not given by any of the two groups except on item number 15 where ‘uncertain’ response was given by 31% of the patients and 36% of the relatives' population. In both these groups, more than 54% of the respondents chose the response ‘agreed’ for this item which was factually incorrect.

## DISCUSSION

Good knowledge about and favourable attitude towards electric shock therapy are considered desirable attributes in therapeutic intervention and good outcome.^5^ Existing knowledge and attitudes, therefore, need to be studied. It is desirable to use standardized schedules/questionnaires/rating scales whenever and wherever available. However, in the absence of standardized scales, one has to use a set of items which are likely to throw some light on these two aspects, i.e. good knowledge and favourable attitude towards ECT. The present study was an effort in this direction.

It is usually seen that however good one's intentions might be and however competent one might be in item construction, some items remain ambiguous and need modifications or even rejection. Sometimes experts also are known to differ (having less than 100% agreement). In the present study, the 20 items were constructed by experts with good knowledge and favourable attitude towards ECT in mind but, as expected, there was less than 100% agreement on some items (4 out of 20) with the result that these had to be discarded and only 16 items remained in the final questionnaire to be administered to the patients and relatives. Further, the questionnaire has not been subjected to a systematic study to evaluate or validate its psychometric soundness. However, face validity and content validity were evaluated and were found to be generally satisfactory.

Test taking attitudes and response biases may affect the results and bias any interpretation. These, however, were found to be negligible in the present study. Both the patients and their relatives came from the same socioeconomic and family backgrounds and had more or less similar attitudes. This is interpreted from the observation that the pattern of responses ‘agree’, ‘disagree’ and ‘uncertain’ were similar across the two groups. Similar lack of effect of socioeconomic status was found by a previous study.^10^ The chances of using ‘uncertain’ response as an escape from the situation were also found to be negligible.

Relatively good knowledge was seen on eleven items (defined as correct response by more than 50% of the respondents); and on four of them it was very good knowledge (defined as correct responses by more than 70% of the respondents) while poor knowledge was seen only on one item (defined arbitrarily as correct responses given by less than 20% of respondents). These criteria, by necessity, have to be somewhat arbitrary. They need to be unambiguously defined so that communication is possible and the meaning is understood by most people; researchers are likely to get more or less similar results on attempts at replication.

### Item-wise discussion of the results

*Item 1 (Pregnant women can also receive ECT):* Only a few of the patients (16.8%) and relatives (15.7%) agreed to it implying that majority of the people did not consider ECT to be safe in pregnancy.

*Item 8 (Patients of epilepsy should not be given ECT):* More than 50% persons did not endorse the use of ECT in epileptic individuals. However, there was a significant proportion of patients (31%) as well as relatives (36%) who acknowledged ignorance and uncertainty implying need for education.

*Item 10 (ECT can be given to older persons also):* More than half of the sample endorsed the assertion implying that opinion is divided and nearly 50% of the people lack knowledge.

*Item 16 (ECT should be given only to patients admitted in the ward):* Slightly less than half the sample agreed with this statement implying that opinion is equally divided and knowledge dissemination is warranted.

*Item 9 (ECT is given as a punishment to violent/angry patients):* Only less than 40% subjects supported this statement. Even this, is a large figure implying serious misconception about the ECT.

*Item 4 (ECT is given to only those patients who have little chance of improvement anyway):* This important misconception was shared by about 70% of the sample implying that prescription of ECT is perceived as communication of non-treatability of the condition by any other means.

*Item 13 (If ECT fails in a patient, then no other treatment will succeed):* About one-third of the subjects harboured this belief, which is likely to result in severe resistance in the subjects even when told that ECT is the first treatment of choice in them.

*Item 3 (Use of ECT leads to permanent loss of memory):* This popular belief was endorsed by only about 10% of the subjects implying good knowledge in the majority.

*Item 6 (Use of ECT leads to temporary impairment of memory):* About 60% of the subjects endorsed this view implying that a significant proportion (40%) of people were not aware of temporary impairment of memory due to ECT. This may be partly responsible for the inflated good response to item 3.

*Item 15 (There is no need of investigations before ECT):* Only 10% of the sample endorsed this view. Thus, the majority of the subjects were aware that certain investigations are mandatory before ECT.

*Item 14 (ECT should not be given more than once a week):* This view was supported by about 65% of the subjects. This, however, could simply imply their lack of technical knowledge.

*Item 12 (ECT is painful):* Patients become unconscious during the ECT, which is painless irrespective of whether the procedure is modified or direct ECT. However, the majority (60%) the subjects people in this study thought otherwise.

*Item 5 (Following discovery of new medicines, treatment with ECT should not be done):* More than half of the subjects endorsed this statement implying that there was a tendency in the sample to replace ECT by some ‘wonder drug’.

*Item 7 (Many times, ECT proves to be life-saving):* This important assertion was supported by more than 85% of the sample implying that people admit that ECT does have its role in certain circumstances.

*Item 11 (There is scientific proof favouring the utility of ECT:* Only about one-fourth of the subjects considered ECT to be unscientific implying that a majority of them included ECT among scientifically validated modes of treatment.

*Item 2 (ECT is an inhuman treatment):* Slightly less than two-thirds of the sample disagreed with this statement. However, it is to be noted that the remaining portion of the sample is still large in magnitude and warrants education.

These results show that the patients as well as their relatives have reasonably good knowledge and favourable attitude towards ECT. The findings are comparable with observations of Freeman and Kendell^6^, Goodman *et al.*^19^ and Ramachandra *et al.*^10^ although their samples consisted of patients who had received ECT in the past. It is under-standable that a lay person is afraid of ECT administration to a pregnant woman or an epileptic patient. From the responses to this questionnaire, one can identify the areas of ignorance, misconception and negative attitude in the patients and their relatives and offer remedial measures. The questionnaire can be given to patients and their relatives before administering ECT to find their attitudes and knowledge towards ECT. Misconceptions and negative attitudes can be taken up during pre-ECT counselling which will enhance acceptance of the procedure.

Some limitations of the study are: Use of un-standardized instrument, failure to separate knowledge and attitude measures, sample restricted to the Department, of Psychiatry and inability to use better statistical measures due to sample characteristics.

## CONCLUSIONS

It is good to know that most of the patients and their relatives are well informed about ECT, its effects and drawbacks. With such a study the areas of misinformation can be identified and overcome, e.g. patients can be reassured that ECT can be given to pregnant women and epileptics also and a patient can be given ECT more than once a week. Further, there was good agreement between patients and relatives across various areas of knowledge and attitude towards ECT. However, in the view of the limitations, the results should be interpreted with caution. This study should be treated more as a preliminary exploratory study and further refinement in methodology should help to draw more robust conclusions. The authors are in the process of standardizing the questionnaire for the general population.
